# An approach to manufacturing well‐being milk chocolate in partial replacement of lecithin by the functional plant‐based combination

**DOI:** 10.1002/fsn3.4051

**Published:** 2024-03-10

**Authors:** Harshvardhan Patel, Aarti Bains, Kandi Sridhar, Nemat Ali, Agnieszka Najda, Mansuri M. Tosif, Sanju Bala Dhull, Prince Chawla, Minaxi Sharma, Gulden Goksen

**Affiliations:** ^1^ Department of Food Technology and Nutrition Lovely Professional University Phagwara India; ^2^ Department of Microbiology Lovely Professional University Phagwara India; ^3^ Department of Food Technology, Karpagam Academy of Higher Education (Deemed to be University) Coimbatore India; ^4^ Department of Pharmacology and Toxicology, College of Pharmacy King Saud University Riyadh Saudi Arabia; ^5^ Department of Vegetable and Herbal Crops University of Life Science in Lublin Lublin Poland; ^6^ Department of Food Science and Technology Chaudhary Devi Lal University Sirsa India; ^7^ Department of Applied Biology University of Science and Technology Meghalaya Baridua India; ^8^ Department of Food Technology, Vocational School of Technical Sciences at Mersin Tarsus Organized Industrial Zone Tarsus University Mersin Turkey

**Keywords:** chocolate, guar gum, lecithin, plant gums, polysaccharide

## Abstract

Lecithin is constituted of a glycerophospholipid mixture and is abundantly used as an emulsifying agent in various food applications including chocolate production. However, overconsumption of lecithin may create an adverse effect on human health. Thus, this study aims to replace the lecithin with plant‐based gums. Different ratios of guar and arabic gum (25%–75%) and their blend (25%–75%) were employed as partial replacement of lecithin. Milk chocolate prepared using 40% guar gum (60GGL [guar gum, lecithin]), 25% arabic gum (75AGL [arabic gum, lecithin]), and a blend of 15 arabic gum and 10 guar gum (65AGGL [arabic gum, guar gum, lecithin]) showed similar rheological behavior as compared to control chocolate (100% lecithin). The fat content of 65AGGL (37.85%) was significantly lower than that of the control sample (43.37%). Rheological behavior exhibited shear‐thinning behavior and samples (60GGL‐75GGL‐80GGL, 65AGL‐75AGL, and 65AGGL‐75AGGL) showed similar rheological properties as compared to control. The chocolate samples (60GGL and 65AGGL) showed significantly (*p* < .05) higher hardness values (86.01 and 83.55 N) than the control (79.95 N). As well, gum‐added chocolates exhibited higher thermal stability up to 660°C as compared to the control sample. The Fourier transform infrared spectroscopy (FTIR) analysis revealed predominant β‐(1 → 4) and β‐(1 → 6) glycosidic linkages of the gums and lecithin. Sensory evaluation revealed a comparable score of gum‐added milk chocolate in comparison to control samples in terms of taste, texture, color, and overall acceptance. Thus, plant exudate gums could be an excellent alternative to lecithin in milk chocolate, which can enhance the textural properties and shelf life.

## Graphical abstract

In this study, guar and arabic gum were utilized as lecithin replacements in the formulation of milk chocolate and their effect on the quality was determined. Herein, different concentrations of guar and arabic gum (25%–75%) and their combination (25%–75%) were used to replace the lecithin. Sensory evaluation revealed a comparable score of gum‐added milk chocolate in comparison to control samples in terms of taste, texture, color, and overall acceptance. Thus, plant exudate gums could be an excellent alternative to lecithin in milk chocolate, which can enhance the textural properties and shelf life.

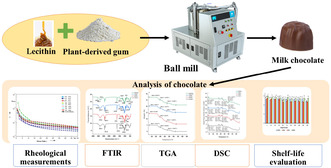

## 1 INTRODUCTION

Lecithin is a lipid mixture comprising various components, such as phospholipids, sterols, glycolipids, fatty acids, and triglycerides. It can be extracted from several sources including plant‐based, animal‐based, and marine. It is commonly used as an emulsifier in the food and confectionary industry to stabilize and homogenize liquid mixtures (Szuhaj et al., 2020). It can be added to chocolate and confectionery products in various forms (liquid and powder). In the production of chocolate, it is typically added during the conching process, where the chocolate is mixed for several hours to develop its texture and flavor. Also, lecithin can be used for the development of various food products, such as hard candies, soft candies, lollipops, and gummies, to improve their texture and appearance (Robert et al., 2021). However, overconsumption of calorie‐dense fat is highly responsible for several chronic diseases including coronary heart disease and obesity. There is a need to find alternative ingredients to replace lecithin due to concerns about food allergies and higher production costs. For example, lecithin possesses various limitations including susceptibility to microbial growth, multifunctionality, and efficiency. Moreover, it has been believed that pregnant and breastfeeding women should avoid consuming lecithin due to its adverse side effects like stomach pain and higher cholesterol levels (Patel et al., 2024; Robert et al., 2021). Also, lecithin contains high calories and can lead to adverse effects, such as nausea, vomiting, diarrhea, and a fishy body odor (List, 2015). Based on the aforementioned reasons, the attraction of plant‐derived polymers like gums has been increasing as an alternative to lecithin. It has been believed that plant‐derived gums can be effectively used for lecithin replacement due to their remarkable water‐holding and emulsifying properties. Thus, replacing lecithin with plant‐based gums could be a great initiative for developing gum‐based novel food products.

Gums are a type of edible carbohydrate biomolecules that exhibit water‐soluble properties and possess water‐holding and gel‐forming abilities due to the presence of hydrophilic and hydrophobic groups (George et al., 2019). Thus, they are widely used in different industrial applications. Furthermore, one of the most important properties of gums are water‐binding capabilities, as well as their capacity to alter and enhance food rheological properties, and their ability to form films or gels, in addition to encapsulating diverse compounds, such as bioactive compounds, flavors, and nutraceuticals (Samakradhamrongthai et al., 2022). Guar gum is a galactomannan polysaccharide derived from the seeds of the guar plant, while arabic gum is a natural exudate from the arabic tree. Due to its excellent water‐binding capacity and rheological properties, it is widely used as a thickening and stabilizing agent in various food products, such as dairy, bakery, and confectionery products (Jaafar, 2019). Arabic gum is widely used as an emulsifier, stabilizer, and thickener in various food applications due to its high solubility and low viscosity (Moghal & Vydehi, 2021). Based on these functionalities, gums are used as additives in sauces, ketchup, confectionery, baby foods, salad dressings, sauces, soups, icings, cake mixes, ice creams, coffee whiteners, cured meat foods, fruit drinks, and beverages (Jaafar, 2019). Both guar gum and arabic gum have been shown to have emulsifying properties, making them promising alternatives to lecithin. In addition, these gums are natural and nontoxic, making them safe for human consumption. They also have other potential health benefits, such as improving digestive health and reducing inflammation (Hu et al., 2016).

Lecithin is derived from various origins and distinct tastes that can alter the quality of traditional milk chocolate. Soybean‐derived lecithin is not healthier enough because it is often extracted using chemicals or solvents like hexane. These solvents may show an adverse effect on human health. Additionally, from a health perspective, lecithin contains phospholipids, which could be problematic for consumers with specific dietary restrictions or allergies (Saputro et al., 2019). Consuming foods containing soy‐based lecithin may lead to symptoms, such as gastrointestinal discomfort, bloating, gas, or digestive disturbances. Few studies have proven that overconsumption of lecithin may cause various diseases including obesity and diabetes (Kaur et al., 2017). On the other hand, it is high in cost, therefore, there is a need to replace the lecithin with plant‐based eco‐friendly and cost‐effective material. Considering its cost‐effectiveness, the use of lecithin in white chocolate production generally proves to be a financially viable choice, as it can contribute to improved production efficiency and reduced ingredient costs. In our previous study (Patel et al., 2024), we studied the effect of guar gum and Arabic gum on the overall quality of the white chocolate. The result revealed the comparable characteristic of gum‐added white chocolate as of the control sample. However, in this study, we explored and optimized the different ratios of guar gum and arabic gum, and their combination was employed for the partial replacement of lecithin in milk chocolate. Also, its effect on the rheological behavior, physicochemical properties, characterization, and shelf life was studied.

## 2 MATERIALS AND METHODS

### 2.1 Materials

#### 2.1.1 Raw materials for milk chocolate formulation

All the food‐grade ingredients including alkalized cocoa powder (Golden Harvest Cocoa, Indonesia), hydrogenated vegetable fat (Bunge India Pvt. Ltd., Mohali, Punjab, India), sugar (Dharmesh Enterprise, Navsari, Gujarat, India), milk powder, skimmed milk powder (SMP) (Amul, Anand, Gujarat, India), liquid soy lecithin (VK Enterprise, Ahmedabad, Gujarat, India), polyglycerol polyricinoleate (PGPR) (Jayant Agro‐Organics Limited, Vadodara, Gujarat, India), guar gum, Arabic gum (Yes Jee Chemicals and Consultants, Mumbai, Maharashtra, India), chocolate flavor (Symega Food Ingredients Limited, Kochi, Kerala, India), and common salt were used to formulate chocolates.

#### 2.1.2 Chemicals

Several analytical‐grade chemicals and reagents including petroleum ether, sodium hydroxide, sodium carbonate, and quercetin were procured from Loba Chemie Pvt. Ltd., Mumbai, India. Sulfuric acid, silver nitrate, Folin–Ciocalteu reagent, citric acid, sodium nitrate, aluminum chloride, ammonium hydroxide, and phenolphthalein indicator were procured from Central Drug House (CDH), New Delhi, India. Ethanol (99.99%) was procured from Changsu Hongsheng Fine Chemical Co. Ltd, China. The research work was conducted using class ‘A’‐certified acid‐washed glassware. Triple distilled water was used to formulate all the required reagents and chemicals.

### 2.2 Methodology

#### 2.2.1 Preparation of milk chocolate mix

The milk chocolates were prepared by flowing the two‐step process according to the method proposed by (Razavizadeh & Tabrizi, 2021) with certain modifications. The chocolate mix was prepared using a ball mill, and the amounts of the colloidal components were optimized based on the comparable rheological behavior of the chocolate mix containing 100% lecithin. In the first step, hydrogenated vegetable fat (28%) was melted in a fat melting tank (Memak, MKYE 1000, Konya, Turkey) at 55°C and other ingredients, such as sugar (45%), cocoa powder (18%), milk powder (13%), skimmed milk powder (SMP) (7%), polyglycerol polyricinoleate (PGPR) (0.2%), and lecithin (0.3%), were mixed with hydrogenated vegetable fat in a mixing tank (Memak, MKYE 1000, Konya, Turkey) for 20 min to ensure a uniform blend. The whole blend was then transferred to the ball mill (Memak, chocolate ball mill, Konya, Turkey) and mixed properly for 3 h at 60°C by with the rotational speed of the ball mill at 250 rpm. For lecithin replacement in chocolate, different concentrations of guar gum (25%–75%), arabic gum (25%–75%), and the blend of guar and arabic gum (25%–75%) were used, as shown in Table 1, respectively. All the samples were added to the chocolate mix during the ball mill processing (after 2.5 h and further mixing was continued for 30 min). Chocolate mix with 100% colloidal components (gums) was not prepared as they caused excessive stickiness, which resulted in damage to the ball mill. Therefore, the prepared milk chocolate mix was then further analyzed for its rheological properties. In the second step, the rheological behavior of the chocolate mix samples was evaluated according to the method proposed by (de Souza Correia Cozentino et al., 2022; Lim et al., 2021) using a cup‐and‐bob type rheometer (Anton Paar, MCR 52, Austria). Herein, the chocolate mix was placed into the sample stand, and the measurement cycle was initiated with a pre‐shear rate of 5 s^−1^ for 10 min at 42°C. Then, after 3 min, the shear rate was gradually increased from 2 to 50 s^−1^. The shear rate of 50 s^−1^ was maintained constant for 1 min, and finally, it was reduced back to 2 s^−1^.

**TABLE 1:** Substitution/replacement of lecithin in milk chocolate.

Ingredients (% w/w)											
Arabic gum‐added milk chocolate	Control	50AGL	55AGL	60AGL	65AGL	70AGL	75AGL	80AGL	85AGL	95AGL	AGL
Sugar	40	40	40	40	40	40	40	40	40	40	40
Hydrogenated vegetable fat	20	20	20	20	20	20	20	20	20	20	20
Cocoa mass	18	18	18	18	18	18	18	18	18	18	18
Milk powder	13	13	13	13	13	13	13	13	13	13	13
SMP	7	7	7	7	7	7	7	7	7	7	7
L: AG	100:0	50:50	55:45	60:40	65:35	70:30	75:25	80:20	85:15	95:05	0:100
PGPR	0.2	0.2	0.2	0.2	0.2	0.2	0.2	0.2	0.2	0.2	0.2
Chocolate flavor	0.6	0.6	0.6	0.6	0.6	0.6	0.6	0.6	0.6	0.6	0.6

Guar gum‐added milk chocolate	50GGL	55GGL	60GGL	65GGL	70GGL	75GGL	80GGL	90GGL	95GGL	GGL
Sugar	40	40	40	40	40	40	40	40	40	40
Hydrogenated vegetable fat	20	20	20	20	20	20	20	20	20	20
Cocoa mass	18	18	18	18	18	18	18	18	18	18
Milk powder	13	13	13	13	13	13	13	13	13	13
SMP	7	7	7	7	7	7	7	7	7	7
L: GG	50:50	55:45	60:40	65:35	70:30	75:25	80:20	90:10	95:05	0:100
PGPR	0.2	0.2	0.2	0.2	0.2	0.2	0.2	0.2	0.2	0.2
Chocolate flavor	0.6	0.6	0.6	0.6	0.6	0.6	0.6	0.6	0.6	0.6

Arabic and guar gum blend added milk chocolate	Control	50AGGL	55AGGL	60AGGL	65AGGL	70AGGL	80AGGL	85AGGL	95AGGL	AGGL
Sugar	40	40	40	40	40	40	40	40	40	40
Hydrogenated vegetable fat	20	20	20	20	20	20	20	20	20	20
Cocoa mass	18	18	18	18	18	18	18	18	18	18
Milk powder	13	13	13	13	13	13	13	13	13	13
SMP	7	7	7	7	7	7	7	7	7	7
L: AGG	100:0	50:25:25	55:25:20	60:20:20	65:20:15	70:15:15	80:10:10	85:7.5:7.5	95:2.5:2.5	0:50:50
PGPR	0.2	0.2	0.2	0.2	0.2	0.2	0.2	0.2	0.2	0.2
Chocolate flavor	0.6	0.6	0.6	0.6	0.6	0.6	0.6	0.6	0.6	0.6

Abbreviations: L: AG, Lecithin: Arabic gum; L: AGG, Lecithin: Arabic and guar gums; L: GG, Lecithin: guar gum; PGPR, Polyglycerol polyricinoleate; SMP, Skimmed milk powder.

#### 2.2.2 Preparation of milk chocolate

Milk chocolate was formulated using the single‐shot continuous molding line connected with a cooling chamber. All the chocolates were cooled at room temperature (28°C) and then transferred to polycarbonate molds. Then, the molds were placed into the cooling chamber (10°C) for 40 min. Following the cooling phase, the chocolates were carefully taken out of the molds, with each sample containing 10 g of material. Samples were hand‐wrapped with aluminum foil and kept until additional analysis, such as proximate, hardness, and energy, and stored at refrigeration conditions (4–7°C) for 150 days.

#### 2.2.3 Proximate analysis and energy value

The chocolate samples were analyzed for their ash, moisture, protein, and fat content by following Association of Official Analytical Chemists (AOAC) methods. The moisture content (g water/100 g dm) was determined by drying a 2 g sample at 105°C using a hot air oven until a constant weight was achieved. To measure the ash content (g ash/100 g dm), the charred chocolate samples were kept in a muffle furnace at 550°C for 6 h. The Kjeldahl method was used to analyze the protein content (g protein/100 g dm). To calculate the fat content (g fat/100 g dm), weight loss was measured after six cycles of extraction with petroleum ether using a Soxhlet apparatus. The carbohydrate content was determined by subtracting the moisture, lipid, protein, and ash contents. To determine the energy content of the chocolate samples, the method proposed by (Saputro et al., 2019) was followed using a Bomb calorimeter (IK‐211, Ikon instruments, New Delhi, India). Herein, 1 g of each chocolate sample was combusted within the calorimeter, which enabled us to measure the heat produced by the combustion of the sample. The energy value was expressed in units of calories per 100 g of chocolate, and the measurement was repeated three times to ensure accuracy. The bomb calorimeter was calibrated with a standard reference material before each measurement, and a blank measurement was performed to account for any background heat.

#### 2.2.4 Hardness measurement

The hardness of milk chocolate samples was measured according to (Ozer et al., 2022) using a Texture Analyzer (TA. XT plus Texture Analyzer, Stable Micro System, United Kingdom). The analysis was conducted using a single penetration event method, with the sample hardness measured at a temperature of (22 ± 1°C). The method involved utilizing a three‐point bending ring (HDP/3 PB) with a span of 40 mm between the supports to test the samples. The analyzer setting was used at 1.0 mm/s speed for the pretest, 3.0 mm/s speed for the test, 10.0 mm/s speed for the posttest, a distance of 10 mm, and a data acquisition rate at 500 pps. The load cell with a capacity of 50 kg was used to measure the force applied to break the sample and the corresponding distance. These measurements were used to determine the hardness of the milk chocolate.

#### 2.2.5 Characterization of milk chocolate

##### Fourier transform infrared spectroscopy (FTIR)

The FTIR spectra of the chocolate were measured, as described by (Deus et al., 2021). The milk chocolate samples underwent pulverization and sieving (with a diameter of 0.149 mm) before being analyzed by an FTIR spectrophotometer (Perkin Elmer, Spectrum Two). To generate the results, a 5 mg sample was placed on the clean mirror surface, and a lens was placed over it. Spectra were then captured within the mid‐infrared range (4000–400 cm^−1^), using air as the background, and transmittance data were produced using the Spectrum 10 software built into the instrument.

##### Differential scanning calorimeter (DSC)

To analyze the thermal properties of the milk chocolate samples by differential scanning calorimeter (PerkinElmer, Waltham, MA, USA), the approach proposed by (Kaur et al., 2021) was used. The analysis was conducted under standard ambient conditions of 25°C temperature and 30% relative humidity. Briefly, 15 mg of sample was placed in a sealed aluminum pan and subjected to a constant rate of heating at 5°C/min, while maintaining a constant flow of nitrogen, from 0 to 70°C. The recorded data were generated at temperature ranges between 10 and 450°C, with a scan rate of 10°/min. Temperature measurements were detected and plotted using thermocouple‐based sensors.

##### Thermogravimetric analysis (TGA)

The thermogravimetric investigation was conducted using a Discovery TGA Analyzer from TA Instruments (PerkinElmer, Waltham, MA, USA) and the technique proposed by (Kaur et al., 2017). The milk chocolate was placed in a platinum container. The experiment was carried out by subjecting the sample to temperatures ranging from 30 to 900°C at a heating rate of 10°C/min, under both nitrogen and oxygen atmospheres, with a flow rate of 25 mL/min. The temperature dependence of mass loss was determined by collecting thermogravimetric (TG) curves, and the first derivative (Derivative Thermography [DTG]) was subsequently derived.

#### 2.2.6 Shelf‐life evaluation

##### pH and titratable acidity

The pH and titrable acidity of milk chocolate were determined as per the methodology described by (Kaur et al., 2017). Briefly, the chocolate sample (1 g) was dispersed with distilled water (10 mL) and the electrode of a digital pH meter (Labtronics LT‐49, Mumbai, India) was lowered into the sample to measure its pH. To determine the titratable acidity of milk chocolate, 1% of the sample was prepared using distilled water, and the resulting solution was titrated with 0.1 N NaOH solution using phenolphthalein as an indicator. The titratable acidity was then calculated using a formula and expressed in units of percentage of lactic acid. To ensure accuracy, the NaOH solution was standardized, and the titration was performed in triplicate.

##### Color measurement

The HunterLab colorimeter (ColorFlex EZ, Murnau, Germany) is used to measure the color of the milk chocolate (Ratrinia et al., 2022). Using the CIELAB system, L*, a*, and b* values can be obtained. Chroma measures the intensity or saturation of a color, while the whiteness index measures the degree of whiteness or lightness. By using these color parameters, a standardized and quantitative assessment of milk chocolate color was obtained. This information can be used to compare different samples, identify color variations or defects, and optimize processing conditions for consistent and high‐quality milk chocolate products.

#### 2.2.7 Sensory evaluation

The sensory evaluation of milk chocolate samples was performed by a group of 30 semi‐trained sensory panelists. The evaluators consisted of both males and females between the ages of 25 and 45. To reduce any potential bias, the chocolate samples were presented on serving dishes labeled with anonymous five‐ to six‐letter codes. To minimize any carryover effects, judges were instructed to cleanse their palates with water between each sample evaluation. Sensory characteristics, such as flavor, mouthfeel, color, texture, and overall acceptability, were assessed using a 9‐point hedonic scale. The study was reviewed and approved by the Institutional Review Board, Lovely Professional University, India. The verbal informed consent was obtained from each participant, prior to their voluntary participation in the study. Sensory assessments were performed in a dedicated laboratory following ISO 8589 specifications for standardized evaluation.

### 2.3 Statistical analysis

All the experiments were conducted and the results were expressed as mean ± standard deviation. One‐way analysis of variance (ANOVA) and two‐way ANOVA were employed to determine significant differences among the samples, using SPSS (SPSS Inc., Chicago, IL, USA). Duncan's multiple range test was used to analyze the effect of storage time and treatment at the significant level of *p* < .05.

## 3 RESULTS AND DISCUSSION

### 3.1 Rheological measurements

**FIGURE 1:**
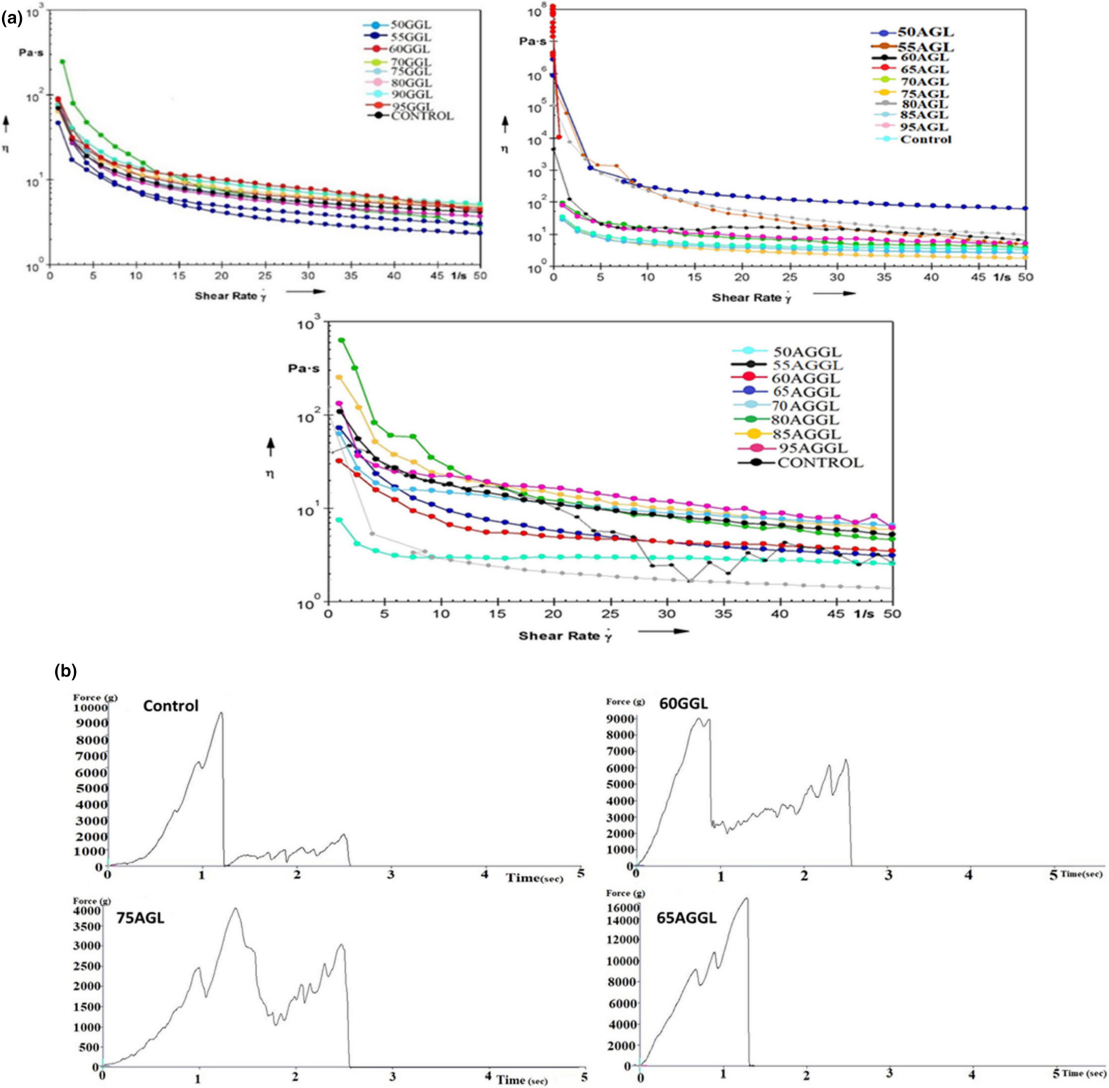
Rheological and textural properties of milk chocolate: (a) Effect of shear rate and temperature on the viscosity of milk chocolate containing guar gum (GGL: guar gum, lecithin), arabic gum (AGL: arabic gum, lecithin), and guar gum and arabic gum (AGGL: arabic gum, guar gum, lecithin), and (b) hardness of Control, 60GGL, 75AGL, and 65AGGL.

The rheological properties of samples are represented in Figure 1a. With increasing shear rates, the viscosity of samples 85GGL, 90GGL, 95GGL, 80AGL, 85AGL, 90AGL, 95AGL, 90AGGL, and 95AGGL was found to be higher than that of the control sample. Whereas, samples 60GGL, 65GGL, 70GGL, 75GGL, 80GGL, 65AGL, 70AGL, 75AGL, 65AGGL, 70AGGL, and 75AGGL showed comparable viscosity to the control sample. However, with increasing shear rates, 50GGL, 55GGL, 50AGL, 55AGL, 60AGL, 50AGGL, 55AGGL, and 60AGGL samples showed lower viscosity as compared to the control sample. The viscosity of the chocolate mix samples (85GGL‐95GGL, 80AGL‐95AGL, and 90AGGL‐95AGGL) was increased due to phase inversion attribution by a high concentration of lecithin and less concentration of gum, resulting in the formation of a stable emulsion system within the chocolate mix. Gums are highly hydrophilic and have high molecular weight. They have a bilayer structure with a hydrophobic tail that interacts with cocoa butter, while the hydrophilic sites interact with water and milk solids (Gavahian et al., 2018). The predominant force on these interactions was the adhesion force between the sugar molecules; however, lecithin reduced this force by the interaction of hydrophilic phosphatidyl groups with the sucrose. On the other hand, the fatty acids of lecithin interacted with the continuous fat phase, which ultimately resulted in increased viscosity of the chocolate mix (Milkova‐Tomova et al., 2019). While, the decrease in the apparent viscosity of the sample (50GGL‐55GGL, 50AGL‐60AGL, and 50AGGL‐60AGGL) with the increased shear rate led to shear‐thinning behavior, which was mainly attributed to the electrostatic interaction between the hydrophobic tails and the carboxyl groups of both gums. In addition, the hydrophilic heads of lecithin interacted with the hydroxyl groups of gums, afterward they interacted with the water and milk solids. The linear chains of mannose and galactose in guar gum and branched chains of arabinose and galactose in Arabic gum allow both colloidal components to absorb a significant number of water molecules, forming a gel‐like network, increasing the thickening and stickiness of the milk chocolate, which caused a delay in ball mill processing and damage to the machine (Middendorf et al., 2016). It was also observed that the viscosity of the samples (60GGL‐75GGL‐80GGL, 65AGL‐75AGL, and 65AGGL‐75AGGL) was similar to that of the control sample. Thus, hydrocolloids such as guar and arabic gum, which are not typically considered strong galactic active agents, improved emulsifying stability primarily by comparing the viscosity of the chocolate mix. Samples prepared using a concentration of gum (25%–40%) interacted with the hydrophilic and hydrophobic side chains of lecithin, forming an emulsifier property that was comparable to that of lecithin. The negatively charged carboxyl group of the gum and the positively charged choline group of the lecithin form a bond, further potentially contributing to the emulsifying properties of the chocolate. Therefore, this interaction of gum and lecithin is a good emulsifier combination, which is effective in stabilizing milk chocolate. It could be inferred that with 40% guar gum and 25% Arabic gum, the blend of both gums (15% GA, 10% GG) was suitable to replace the lecithin and to avoid the stickiness in ball mill processing. Our results are well supported by the findings of (Sun et al., 2021) who reported a similar trend in the rheological behavior of milk compound chocolate using a different concentration of γ‐oryzanol/lecithin/stearic acid.

### 3.2 Proximate analysis and energy value

The proximate analysis of milk chocolate samples is represented in Table 2. The moisture content of chocolate plays a vital role in determining its texture and shelf life. In the study, all the samples, that is, 60GGL (1.59 ± 0.03 g/100 g), 75AGL (1.64 ± 0.17 g/100 g), and 65AGGL (1.68 ± 0.58 g/100 g) showed nonsignificant (*p* > .05) difference in moisture content as compared to the control sample (1.75 ± 0.13 g/100 g). These findings were consistent with the results reported by (Razavizadeh & Tabrizi, 2021) where milk chocolate enriched with microencapsulated chia seed oil exhibited a moisture content of 0.9%–1.05%. Furthermore, the ash content of the chocolate samples was in the range of 0.98 ± 0.03 to 1.17 ± 0.11 g/100 g and all the samples showed nonsignificant (*p* > .05) differences as compared to the control chocolate sample. In addition, all the chocolate samples showed significant differences in terms of protein content; however, 60GGL (6.22 ± 0.2 g/100 g) and 75AGL (6.41 ± 0.05 g/100 g) showed significant (*p* < .05) differences with each other. Whereas 65AGGL (5.98 ± 0.01 g/100 g) showed nonsignificant higher protein content than the control sample (5.61 ± 0.11 g/100 g). Higher protein content in chocolate samples was mainly attributed to the significant protein content of the milk powder and gums, respectively. Our results were well correlated according to a study conducted by (n.d.‐b) and (Saunshia et al., 2018) who revealed that milk chocolate has 3.5–6.75 g/100 g protein and 0.8–1.6 g/100 g ash content, respectively, due to the presence of minerals, such as potassium, calcium, magnesium, and iron. Also, the control chocolate sample showed significantly higher fat content, whereas 65 AGGL (37.85 ± 0.49 g/100 g) showed significantly lower fat content as compared to the other counterparts. However, the fat content of the milk chocolate samples 60GGL (41.17 ± 0.71 g/100 g), and 75AGL (42.98 ± 0.32 g/100 g) was found to be nonsignificant (*p* > .05) in comparison to the control sample (43.37 ± 1.65 g/100 g). The lower fat content of 65AGGL was mainly attributed to a 35% replacement of lecithin as compared to other chocolate samples. Our results were consistent with the findings of Bursa et al. (2021) who revealed 32.0 g/100 to 45.0 g/100 g fat content of the milk chocolate. Moreover, the milk chocolate samples of content gum showed a significant amount in terms of crude fiber; however, 60GGL (1.62 ± 0.02 g/100 g), 75AGL (1.54 ± 0.01 g/100 g), and 65AGGL (1.75 ± 0.02 g/100 g) showed significant differences in compression with each other. The carbohydrates of the milk chocolate sample, that is, 75AGL (47.88 ± 0.03 g/100 g) showed nonsignificant differences compared with the control sample (48.1 ± 0.01 g/100 g). However, 60GGL (50.04 ± 0.02 g/100 g) and 65AGGL (53.45 ± 0.02 g/100 g) were significantly higher compared to the control sample. Higher carbohydrate in chocolate samples was mainly attributed to the significantly lower amount of fat present in that sample. Moreover, the energy value of milk chocolate was analyzed after the addition of guar and arabic gum as a lecithin replacer. In the study, all the samples, that is, 60GGL (515.2 kcal/100 g), 75AGL (508.2 kcal/100 g), and 65AGGL (501.2 kcal/100 g) showed significant differences (*p* > .05) in energy value as compared to the control sample (498.3 kcal/100 g). Moreover, these results suggested that replacing lecithin with guar and arabic gum could significantly impact the energy value of milk chocolate, which was due to the high carbohydrate content of arabic gum and guar gum as compared to lecithin. This could be attributed to the higher molecular weight and higher solubility of arabic gum compared to guar gum, resulting in a more significant increase in the energy value (Verde et al., 2021). The results revealed that the addition of both gums had led to an increase in energy value but guar gum showed a greater effect than arabic gum.

**TABLE 2:** Proximate composition of milk chocolate^a^.

Sample	Moisture (g/100 g)	Ash (g/100 g)	Fat (g/100 g)	Protein (g/100 g)	Crude fiber (g/100 g)	Carbohydrates (g/100 g)	Energy value (kcal/100 g)
60GGL	1.59 ± 0.03^a^	0.98 ± 0.03^a^	41.17 ± 0.71^b^	6.22 ± 0.01^c^	1.62 ± 0.02^a^	50.04 ± 0.02^b^	515.2 ± 0.25^d^
75AGL	1.64 ± 0.17^a^	1.09 ± 0.05^a^	42.98 ± 0.32^b^	6.41 ± 0.05^c^	1.54 ± 0.01^a^	47.88 ± 0.03^a^	508.2 ± 0.42^c^
65AGGL	1.68 ± 0.58^a^	1.04 ± 0.05^a^	37.85 ± 0.49^a^	5.98 ± 0.01^b^	1.75 ± 0.02^a^	53.45 ± 0.02^c^	501.2 ± 0.18^b^
CONTROL	1.75 ± 0.13^a^	1.17 ± 0.11^a^	43.37 ± 1.65^b^	5.61 ± 0.11^a^	ND	48.1 ± 0.01^a^	498.3 ± 0.42^a^

*Note*: Mean values within a column with different lowercase superscripts (a–d) are significantly different (*p* < .05) from each other based on Duncan's multiple range test.

Abbreviation: ND, not detected.

^a^ Data are presented as mean ± SD (*n* = 3).

### 3.3 Hardness measurement

The hardness of the milk chocolate containing guar gum, arabic gum, and lecithin is shown in Figure 1b. The milk chocolate sample including 75AGL (79.87 ± 0.50 N) showed a nonsignificant (*p* > .05) difference compared to the control sample (79.95 ± 2.92 N). However, the samples 60GGL (86.01 ± 1.43 N) and 65AGGL (83.55 ± 0.73 N) were significantly different (*p* < .05) from the control samples. The study attributed the significant differences in the use of guar gum are due to its high water‐absorbing capacity and ability to create strong gels that increase the viscosity compression of milk chocolate. In contrast, arabic gum acts as a stabilizer and emulsifier, affecting the microstructure of the chocolate and potentially influencing its hardness (Raoufi et al., 2012). Additionally, the addition of milk powder to chocolate results in reduced hardness because of the change in the fatty acid composition of the continuous phase due to the milk fat content. The addition of guar and arabic gum as a lecithin replacer in milk chocolate can affect the texture and hardness of the product (Bahari & Akoh, 2018). The higher hardness value of milk chocolate with guar gum and arabic gum is attributed to the different physicochemical properties of these gums compared to lecithin, which is a phospholipid that interacts with both water and fat (Verde et al., 2021).

### 3.4 Fourier transform infrared spectroscopy (FTIR)

**FIGURE 2:**
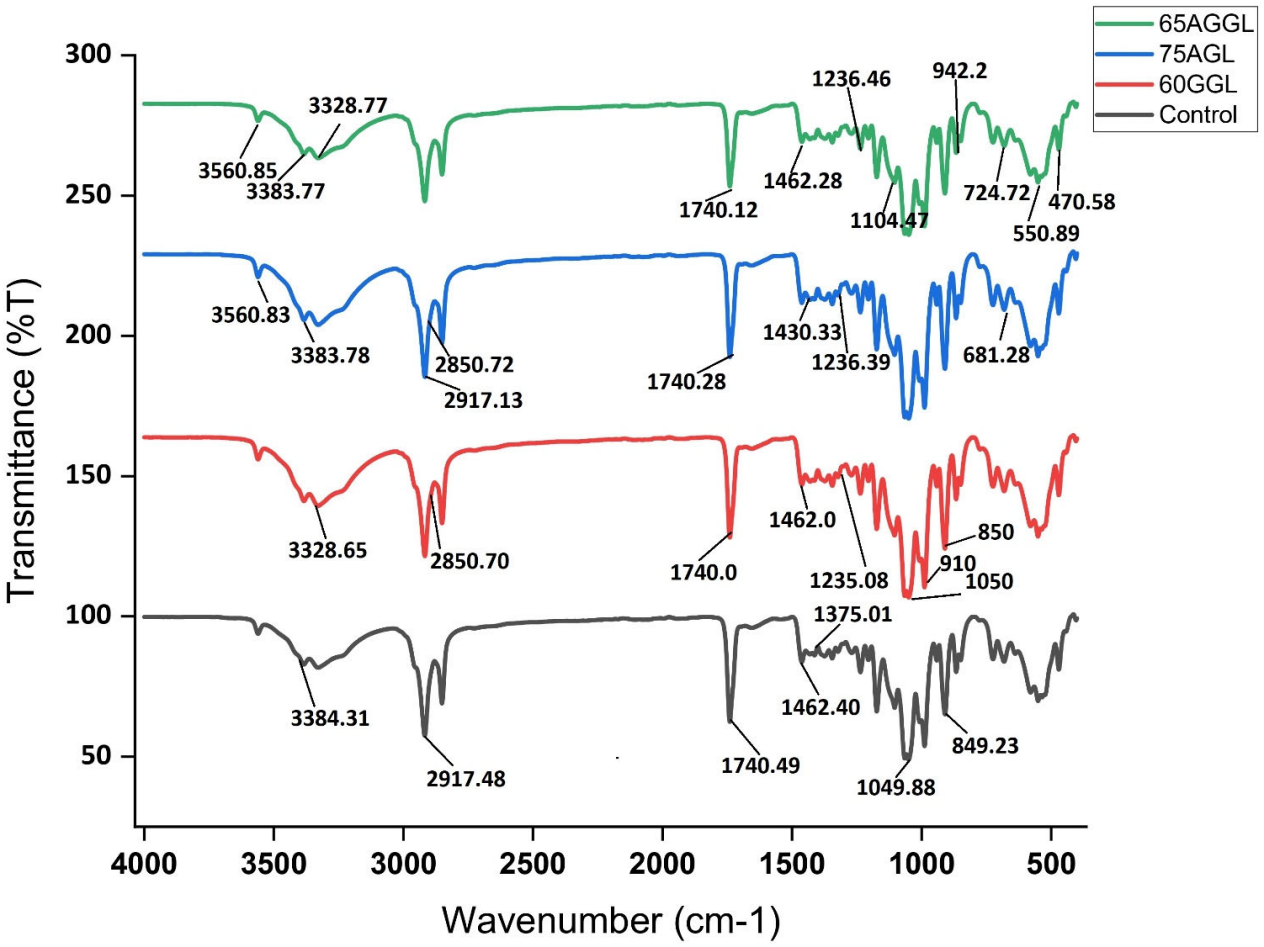
Confirmation of lecithin and colloidal interaction in milk chocolate using FTIR. AGGL, arabic gum, guar gum, lecithin; AGL, arabic gum, lecithin; GGL, guar gum, lecithin.

The FTIR spectrum of the milk chocolate demonstrated the presence of multiple functional groups and the interaction of gums with lecithin, as shown in Figure 2. Herein, the samples 60GGL, 75AGL, and 65AGGL showed several strong absorption bands at 3328.65, 2850.70, and 1462.0 cm^−1^, which were associated with OH, CH, and N‐H stretching, respectively. The peaks around 1070–1030 and 910–850 cm^−1^ confirmed β‐(1 → 4) and β‐(1 → 6) glycosidic linkages of galactose and mannose units of the gums with lecithin. The band is around 1743 cm^−1^, suggesting the absence of ester linkages in gum. The absence of this peak in the presence of gum could indicate changes in the ester linkages due to the different chemical natures of gum. The band observed at 1682 cm^−1^ indicates the decreased hydrogen bond strength and the intermolecular force. This band intensity may slightly vary due to the presence of gum, indicating potential interactions between gum and proteins present in the chocolate matrix. Also, lecithin contains a phosphodiester linkage in its structure that bands around 1235 cm^−1^ corresponding to assessing any changes in the phosphodiester bond due to the added gums. This vibration may be among those that contribute the most to the prediction of spermidine (1022 cm^−1^), cadaverine (1175 cm^−1^), and tyramine (1177 cm^−1^). Therefore, the modified peak indicates a difference in the molecular structure and linkage between the lecithin and gums in milk chocolate. Moreover, a peak at 3384.31 cm^−1^ indicates the stretching vibrations of the hydroxyl (OH) group existing in the sugar and water content of the chocolate. Also, the band observed at 2917.48 cm^−1^ was attributed to the stretching vibrations of the aliphatic CH (methyl and methylene) groups of cocoa butter and milk fat of the chocolate. The band observed at 1740.49 cm^−1^ corresponds to the stretching vibrations of the carbonyl (C=O) groups shown in the milk solid and fat. Similarly, the peak at approximately 1462.40 cm^−1^ indicates the carbonyl (C=O) group in the amide I region, which was analytical of the presence of lecithin. The band observed at around 1375.01 cm^−1^ suggests that the bending vibration of the methyl (CH_3_) group indicates the presence of fatty acids and triglycerides in the milk chocolate. The peak at approximately 1049.88 cm^−1^ reveals the stretching vibrations of the C‐O functional group present in the sugar and cocoa components of the milk chocolate. Our results are comparable with the findings of (Hu et al., 2016) who performed the FTIR for the milk chocolate and revealed similar peaks, including C‐H, O‐H, C=O, and C‐O. Specifically, the peaks at approximately 2916–2925 cm^−1^ (C‐H stretching), 1700–1736 cm^−1^ (C=O stretching), 1520–1540 cm^−1^ (amide N‐H bending), 1050–1230 cm^−1^ (C‐O‐C stretching), 3200–3400 cm^−1^ (O‐H stretching), and 950–980 cm^−1^ (methyl C‐H bending) were consistent with previous studies on chocolate samples.

### 3.5 Differential scanning calorimetry (DSC)

**FIGURE 3:**
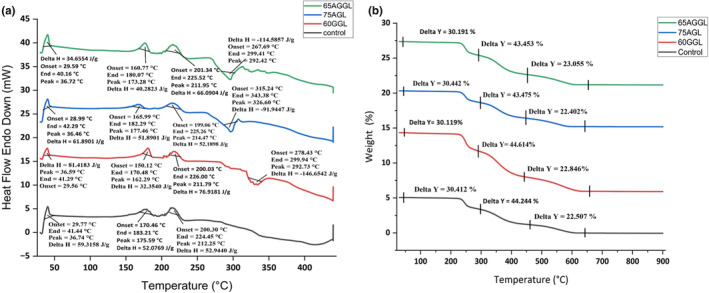
Thermal stability of milk chocolate. (a) DSC thermographs of Control, 60GGL, 75AGL, and 65AGGL, and (b) TGA thermographs of Control, 60GGL, 75AGL, and 65AGGL. AGGL, arabic gum, guar gum, lecithin; AGL, arabic gum, lecithin; GGL, guar gum, lecithin.

Denaturation temperature (Td), enthalpy change (ΔH), and phase transitions are shown in Figure 3a. In the DSC curve, the control sample exhibited an initial endothermic peak after cooling to 15°C. The peak was observed at a peak temperature of 36.74°C with the corresponding fusion enthalpy (ΔH) of 59.3158 J/g. The peak indicated the melting process of the cocoa butter with its specific polymorphic form and milk solids contained within the chocolate. It was the same for all samples including 60 GGL (36.46°C and 81.41883 J/g), 75AGL (36.59°C and 81.41883 J/g), and 65AGGL (36.72°C and 81.41883 J/g). The second endothermic peak of all samples including 60 GGL (177.46°C and 51.8901 J/g), 75AGL (162.29°C and 32.3540 J/g), and 65AGGL (173.28°C and 40.2823 J/g) was compared with that of the control sample detected at 175.59°C and 52.076 J/g. This peak was associated with the decomposition of sugar and the presence of minerals. Similarly, the exothermic peak of all samples including 60GGL (326.60°C and −91.9447 J/g), 75AGL (292.73°C and −146.6542 J/g), and 65AGGL (292.42°C and −114.5857 J/g) was compared with that of the control sample detected at 315.64°C and −125.6745 J/g. This peak was related to the degradation of the samples at high temperatures. The addition of guar and arabic gum as lecithin replacers resulted in a slightly lower onset temperature and peak temperature compared to the control sample, possibly due to the disruption of the crystal structure of cocoa butter and milk solids caused by the addition of gums. This led to a change in their polymorphic form, resulting in a decrease in the onset and peak temperatures. In contrast, the enthalpy of melting was higher in the compound chocolate samples with added gums compared to the control sample (Dolatowska‐Żebrowska et al., 2019). This could be attributed to the higher degree of crystallinity of cocoa butter and milk solids in the compound chocolate samples, requiring more energy to break the bonds holding the crystals together, leading to a higher enthalpy of melting.

### 3.6 Thermogravimetric analysis (TGA)

A thermogravimetric analysis (TGA) of milk chocolate samples is shown in Figure 3b. The TGA curves of the milk chocolate samples exhibited three weight loss phases. The first weight loss, which was reported for 30.41% of the initial weight of the control sample, occurred between 30 and 290°C, indicating the evaporation of moisture and volatile compounds. The second weight loss, which was reported for 44.24% of the initial weight of the control sample, occurred between 290 and 460°C, indicating the decomposition of the sugar and milk solids. The third weight loss, which was reported for 22.51% of the initial weight of the control sample, occurred between 460 and 640°C, indicating the decomposition of the cocoa solids. The overall loss of the sample occurred within 609.33°C, indicating a 100% weight loss. The milk chocolate samples compressed with 60GGL exhibited a first weight loss of 30.12% between 30 and 280°C. The second weight loss was 44.61% between 280 and 440°C, while the third weight loss was 22.85% between 440 and 660°C. The overall loss of this sample occurred within 627.16°C, indicating it to be more heat stable compared with the control sample. Similarly, the 75AGL exhibited a first weight loss of 30.44% between 30 and 280°C, with the second weight loss of 43.48% occurring between 280 and 440°C, and the third weight loss of 22.40% occurring between 440 and 630°C. The milk chocolate samples compressed with a combination of 65AGGL exhibited a first weight loss of 30.19% between 30 and 280°C, with the second weight loss of 43.45% occurring between 280 and 440°C, and the third weight loss of 23.06% occurring between 440 and 660°C. In conclusion, the addition of guar and arabic gum as lecithin replacers resulted in a slight increase in weight loss in the first region, indicating a slight increase in the moisture content of the samples. However, the weight loss in the second and third regions decreased significantly with increasing concentration of the gum additives (Mumbach et al., 2022).

### 3.7 Shelf‐life evaluation

#### 3.7.1 pH and titratable acidity

The shelf life of milk chocolates was observed by measuring their pH and titratable acidity for 150 days at 15‐day intervals (Table 3). Herein, during the 60th day of storage, the pH of all samples including 75AGL (6.31 ± 0.02) and 65AGGL (6.31 ± 0.01) was found to be nonsignificant in comparison to the control sample (6.33 ± 0.03); however, the sample 60GGL (6.32 ± 0.01) showed a significant (*p* < .05) difference as compared to the control sample. Also, during the 75th day, the pH of 65AGGL (6.31 ± 0.01) was found to be nonsignificant in comparison to the control sample (6.31 ± 0.05). In addition, the samples including 60GGL (6.30 ± 0.02) and 75AGL (6.30 ± 0.07) showed a significant (*p* < .05) difference as compared to the control sample. However, a significant (*p* < .05) decline in the pH of all the products over time may be due to the production of formic acid as a result of isomerization and degradation of lactose present in milk powder. Additionally, alterations taking place during storage that promote protein cross‐linking or dephosphorylation of caseins contribute to a decrease in pH (Tolve et al., 2022). Likewise, the titratable acidity of all samples of milk chocolate steadily increased over the 150‐day storage period (Table 3). Herein, during the 60th day of storage, the titratable acidity of 65AGGL (0.17 ± 0.005 w/w lactic acid) showed a nonsignificant difference in comparison to the control sample (0.17 ± 0.008). However, 60GGL (0.16 ± 0.012 w/w lactic acid) and 75AGL (0.16 ± 0.008 w/w lactic acid) were significantly (*p* > .05) different as compared to the control sample. Similarly, during the 75th day, the titratable acidity of 65AGGL (0.18 ± 0.005 w/w lactic acid) showed a nonsignificant (*p* > .05) difference compared to the control sample (0.18 ± 0.008 w/w lactic acid). However, 60GGL (0.17 ± 0.012 w/w lactic acid) and 75AGL (0.16 ± 0.008 w/w lactic acid) were significantly (*p* > .05) different as compared to the control sample. On the other hand, the storage period increased the titratable acidity of all the samples significantly as compared to the control sample. The gradual increase in titratable acidity for all samples over the 150‐day storage period is an indication of the formation of free fatty acids, which can lead to the development of rancid flavor and off‐odors in chocolate (Shashikant et al., 2022). These findings indicated that the addition of different concentrations of gums influenced the stability of the chocolate during the storage period, with the higher concentration of arabic gum in the 75AGL sample contributing to a higher level of titratable acidity. Therefore, it is essential to monitor titratable acidity levels to ensure that milk chocolates remain stable and do not develop undesirable flavors or odors during storage (Tirgarian et al., 2023).

**TABLE 3:** Effect of storage on pH and titratable acidity of milk chocolate at 30 ± 1°C^a^.

	Sample	Storage period (days)										
		0	15	30	45	60	75	90	105	120	135	150
pH	Control	6.33 ± 0.03^aA^	6.33 ± 0.02^aA^	6.33 ± 0.05^aA^	6.33 ± 0.02^aA^	6.33 ± 0.03^aA^	6.31 ± 0.05^aA^	6.30 ± 0.01^aA^	6.29 ± 0.02^bA^	6.29 ± 0.02_bA_	6.28 ± 0.01^bB^	6.27 ± 0.02^aB^
	60GGL	6.32 ± 0.01^gB^	6.32 ± 0.07^gB^	6.32 ± 0.02^gB^	6.32 ± 0.02^gC^	6.32 ± 0.02^gC^	6.30 ± 0.02^fA^	6.29 ± 0.02^eA^	6.28 ± 0.05^dA^	6.27 ± 0.04^cA^	6.26 ± 0.02^bA^	6.25 ± 0.01^aA^
	75AGL	6.31 ± 0.02^eA^	6.31 ± 0.01^eA^	6.31 ± 0.02^eA^	6.31 ± 0.02^eA^	6.31 ± 0.04^eA^	6.30 ± 0.07^dA^	6.30 ± 0.01^dB^	6.29 ± 0.02^cA^	6.28 ± 0.03^bA^	6.27 ± 0.04^bB^	6.26 ± 0.02^aB^
	65AGGL	6.31 ± 0.01^gA^	6.31 ± 0.02^gA^	6.31 ± 0.02^gA^	6.31 ± 0.01^gB^	6.31 ± 0.02^fB^	6.31 ± 0.02^fB^	6.30 ± 0.02^eB^	6.29 ± 0.01^dA^	6.28 ± 0.13^cA^	6.27 ± 0.03^bB^	6.26 ± 0.01^aB^
Titratable acidity (w/w lactic acid)	Control	0.17 ± 0.008^aB^	0.17 ± 0.008^aB^	0.17 ± 0.005^aB^	0.17 ± 0.005^aB^	0.17 ± 0.005^aB^	0.18 ± 0.008^bB^	0.18 ± 0.003^bA^	0.19 ± 0.003^cB^	0.20 ± 0.003^dB^	0.21 ± 0.002^eB^	0.22 ± 0.025^fB^
	60GGL	0.16 ± 0.012^aA^	0.16 ± 0.008^aA^	0.16 ± 0.012^aA^	0.16 ± 0.005^aA^	0.16 ± 0.008^aA^	0.17 ± 0.012^bA^	0.18 ± 0.003^cA^	0.19 ± 0.003^dA^	0.20 ± 0.005^eA^	0.21 ± 0.004^fA^	0.23 ± 0.015^gA^
	75AGL	0.16 ± 0.008^aC^	0.16 ± 0.008^aC^	0.16 ± 0.007^aC^	0.16 ± 0.005^aC^	0.16 ± 0.008^aC^	0.16 ± 0.008^bC^	0.17 ± 0.003^cC^	0.18 ± 0.003^dC^	0.19 ± 0.012^eC^	0.20 ± 0.018^fC^	0.22 ± 0.003^gC^
	65AGGL	0.17 ± 0.005^aB^	0.17 ± 0.008^aB^	0.17 ± 0.005^aB^	0.17 ± 0.005^aB^	0.17 ± 0.008^aB^	0.18 ± 0.005^bB^	0.19 ± 0.005^cB^	0.19 ± 0.003^dB^	0.20 ± 0.003^eB^	0.21 ± 0.001^fB^	0.22 ± 0.015^gB^

*Note*: Mean values within a column with different lowercase superscripts (a–g), and within a row with different uppercase superscripts (A–C) are significantly different (*p* < .05) from each other based on Duncan's multiple range test.

^a^ Data are presented as mean ± SD (*n* = 3).

#### 3.7.2 Color measurement

The color of milk chocolate was affected by various factors, such as the type of cocoa bean and origin, roasting intensity, and the addition of milk and sugar. However, the addition of guar and arabic gum as a lecithin replacer in milk chocolate can affect its color. The study results showed that the mean L* values of the samples, that is, 60GGL (46.45 ± 0.04) and 65AGGL (46.33 ± 0.02) were significantly different (*p* < .05) than the control sample (48.27 ± 0.15); however, 75AGL (49.32 ± 0.50) was nonsignificant from the control sample, indicating a moderately light color. The mean a* values of the samples, that is, 60GGL (8.69 ± 0.05), 75AGL (8.44 ± 0.29), and 65AGGL (8.67 ± 0.03) showed non‐significant differences compared to the control sample (8.24 ± 0.07), indicating a slightly reddish hue. The mean b* values of the sample, that is, 60GGL (17.84 ± 0.12) and 65AGGL (17.64 ± 0.12) were nonsignificant (*p* > .05) than the control sample (17.72 ± 0.24). Moreover, 75AGL (19.96 ± 0.04) showed a significant difference compared to the control sample, indicating a yellowish tone. After 150 days of shelf‐life study, the mean L* values of 60GGL (47.02 ± 0.02), 75AGL (47.94 ± 0.04), and 65AGGL (46.98 ± 0.02) showed nonsignificant (*p* > .05) differences compared to the control sample (49.78 ± 0.03). Moreover, the mean a* values of 60GGL (8.86 ± 0.01), 75AGL (8.86 ± 0.02), and 65AGGL (8.97 ± 0.02) were nonsignificant (*p* > .05) than the control sample (8.73 ± 0.04). Similarly, the mean b* values of 60GGL (18.58 ± 0.02), 75AGL (18.08 ± 0.05), and 65AGGL (18.53 ± 0.07) showed a nonsignificant difference from the control sample (18.09 ± 0.04). On the other hand, the decrease in the L* value and the increase in the a* and b* values with the addition of guar and arabic gum indicate that the chocolate became darker and reddish (Lim et al., 2021). The decrease in the L* value can be attributed to the light‐scattering properties of the gums. Both guar and arabic gum are hydrocolloids that can scatter light and reduce the transparency of the chocolate, resulting in a darker appearance. The increase in the a* and b* values can be attributed to the presence of pigments in the gums. Arabic gum contains anthocyanins and red pigments, while guar gum contains carotenoids and yellow pigments. These pigments can contribute to the reddish and yellowish hue of the chocolate. Similar results were obtained from the study done by (Castro‐Alayo et al., 2023) and revealed that the compound milk chocolate had a lightness value (L*) of around 45–50, indicating a relatively dark color. The a* value was around 6–9, indicating a slightly reddish tint and the b* value was around 18–25, indicating a yellowish tint. In conclusion, the color analysis of milk chocolate incorporating guar and arabic gum, as well as other milk chocolate controls, reveals the potential for these additives to influence the color characteristics of the final product.

### 3.8 Sensory evaluation

**FIGURE 4:**
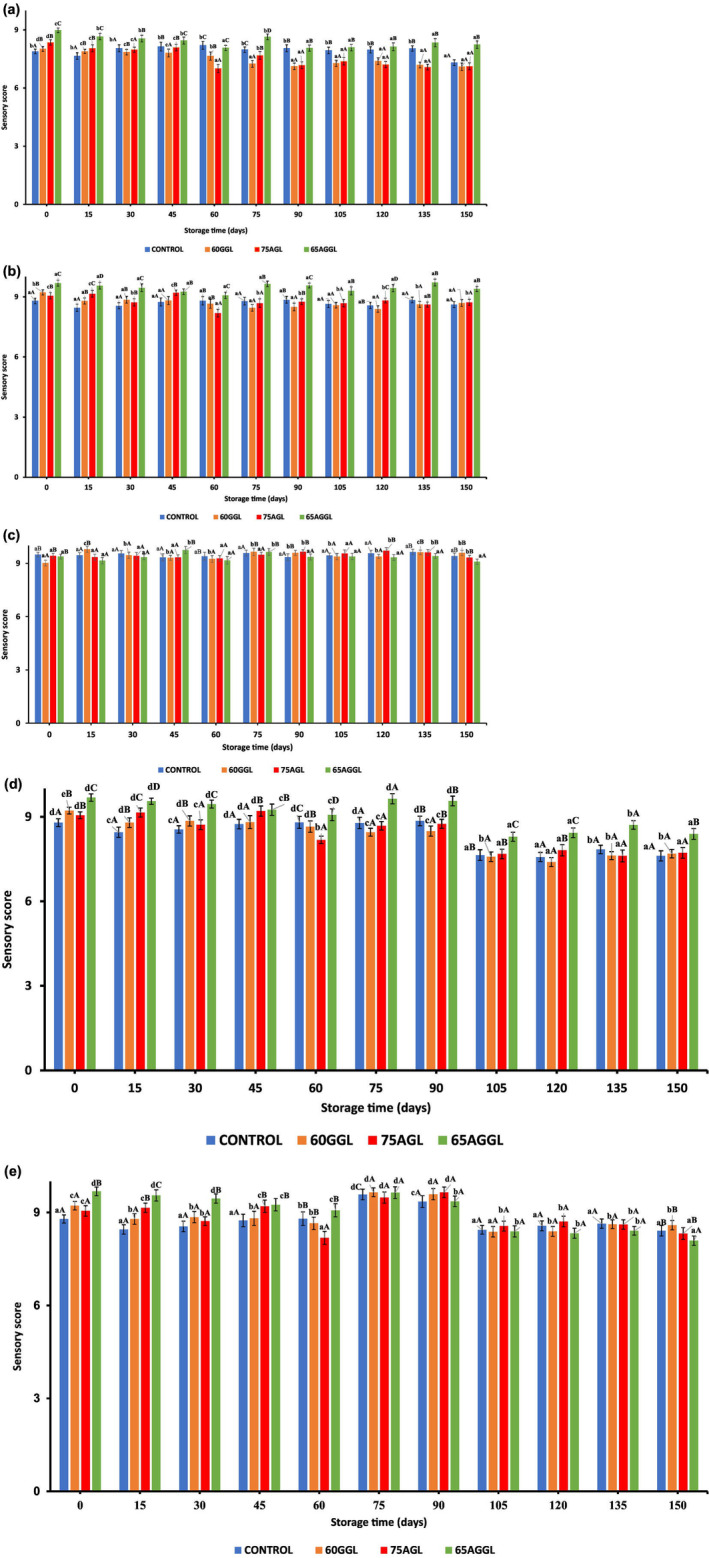
Effect of storage on sensory attributes of milk chocolate's (a) appearance, (b) aroma, (c) texture, (d) flavor, and (e) overall acceptability. AGGL, arabic gum, guar gum, lecithin; AGL, arabic gum, lecithin; GGL, guar gum, lecithin.

The study used semi‐trained experts to evaluate the appearance, aroma, texture, flavor, and overall acceptability of the samples for 15‐day intervals to 150 days, as shown in Figure 4. The sensory scores of all samples exhibited a decreasing trend with storage time across all sensory parameters. Furthermore, the lowest scores were observed after the storage period. The results indicated that the use of guar and arabic gum as lecithin replacers in milk chocolate samples can have a moderate effect on the product's overall acceptability (Mejía et al., 2021). The control samples were the most well‐liked, receiving the highest mean scores for all attributes, while the guar gum replacer lecithin samples were the least liked, receiving the lowest mean scores for all attributes. The arabic gum replacer lecithin samples received slightly higher mean scores than the guar gum replacer lecithin samples, indicating that the use of arabic gum may have a less significant impact on sensory attributes. The mix of guar gum and arabic gum replacer lecithin samples received slightly higher mean scores than the arabic gum replacer lecithin samples, suggesting that the use of a mix of gums may have a slightly less significant impact on sensory attributes. Overall, the study provides valuable information for manufacturers seeking to replace lecithin in their chocolate products while maintaining the desired sensory properties (Batista et al., 2016). It suggests that the type of gum used as a lecithin replacer can significantly impact the product's overall acceptability and should be carefully considered when developing new chocolate products. The results of the sensory evaluation of milk chocolate samples with different lecithin replacers provide valuable insights for manufacturers and researchers in the food industry.

## 4 CONCLUSIONS

The plant‐derived gum replacers utilized in this study exhibited a non‐significant influence (*p* ≥ .05) on the overall characteristics of the milk chocolate. This finding highlights their potential as substitutes for gums in milk chocolate production. Additionally, the gums contributed to enhancing the shelf life of the chocolate and reducing the overall fat content. In terms of sensory evaluation, the chocolate formulations incorporating a blend of plant exudate gums demonstrated a notable disparity in flavor or texture compared to the control sample containing lecithin. The overall acceptability of the chocolate was also comparable to that of the control formulation. Furthermore, the addition of gums can hold the moisture of milk chocolate, due to the fact that the gums decrease the growth of microorganisms and mold development by managing water activity and moisture distribution, extending the shelf life of the milk chocolate. Therefore, the use of a binary blend of plant exudate gums in milk chocolate formulation has the potential to be a promising approach for the development of cost‐effective chocolate products. This study highlights the potential of plant‐based ingredients as a sustainable alternative to conventional ingredients in the food industry. The use of gums such as guar and arabic gum as lecithin replacers in milk chocolate is a promising alternative that offers several advantages over lecithin.

## AUTHOR CONTRIBUTIONS

**Harshvardhan Patel:** Conceptualization (equal); data curation (equal); formal analysis (equal); investigation (equal). **Aarti Bains:** Data curation (equal); investigation (equal); methodology (equal). **Kandi Sridhar:** Investigation (equal); methodology (equal); software (equal). **Nemat Ali:** Funding acquisition (equal); software (equal); validation (equal); writing – original draft (equal); writing – review and editing (equal). **Agnieszka Najda:** Software (equal); visualization (equal); writing original – draft (equal). **Mansuri M. Tosif:** Investigation (equal); methodology (equal); resources (equal); software (equal). **Sanju Bala Dhull:** Data curation (equal); methodology (equal); validation (equal); writing – review and editing (equal). **Prince Chawla:** Investigation (equal); project administration (equal); supervision (equal); writing – original draft (equal). **Minaxi Sharma:** Investigation (equal); methodology (equal); software (equal). **Gulden Goksen:** Software (equal); writing – review and editing (equal).

## CONFLICT OF INTEREST STATEMENT

The authors declare no conflict of interest.

## Data Availability

The data that support the findings of this study are available from the corresponding author upon reasonable request.
